# Regulation of the Total Cell Surface Area in Dividing *Dictyostelium* Cells

**DOI:** 10.3389/fcell.2020.00238

**Published:** 2020-04-08

**Authors:** Masahito Tanaka, Koushiro Fujimoto, Shigehiko Yumura

**Affiliations:** Graduate School of Sciences and Technology for Innovation, Yamaguchi University, Yamaguchi, Japan

**Keywords:** cell division, cell membrane, cytokinesis, endocytosis, exocytosis

## Abstract

When a cell divides into two daughter cells, the total cell surface area should increase. There are two models for membrane supply to support cell division: (1) unfolding of small surface membrane reservoirs such as microvilli or wrinkles and (2) exocytosis of intracellular vesicles. Here, we precisely measured the total cell surface area in dividing *Dictyostelium* cells, flattened by the agar overlay that eliminated the complexity of unfolding surface membrane reservoirs. Because the cells divided normally under the agar overlay, unfolding of surface membrane reservoirs was not required for cell division. Under the agar overlay, the total cell surface area slightly decreased from the interphase to the metaphase and then increased about 20% during cytokinesis. Both endocytosis and exocytosis were suppressed in the early mitotic phase but recovered during cytokinesis. The imbalance of endocytosis and exocytosis could contribute to the changes observed in the cell surface area. Clathrin-dependent endocytosis was also substantially suppressed during cytokinesis, but contrary to previous reports in cultured animal cells, it did not significantly contribute to the regulation of the cell surface area. Furrowing during cytokinesis was indispensable for the cell membrane increase, and vice versa.

## Introduction

During cell division, cells vigorously change shape, and their surface area should therefore be changed accordingly. Assuming that a perfectly spherical cell divides into two perfectly spherical daughter cells, the surface area should increase by 26%. Precise measurement of the total cell surface area is difficult because small microvilli or wrinkles of cell surface, which occupy 21–130% of the apparent cell surface area (Schmid-Schonbein et al., 1980; Guillou et al., 2016), complicate the measurement. Recent measurements using fluorescence exclusion (Cadart et al., 2018), microfabricated channels (Varsano et al., 2017), and lattice light-sheet microscopy (Aguet et al., 2016) have enabled more precise cell volume estimation, but it is still difficult to precisely measure the cell surface area. Nevertheless, previous measurements have shown that the total cell surface area changes during cell division (Boucrot and Kirchhausen, 2007; Giansanti et al., 2015; Aguet et al., 2016).

There are two models to explain the regulation of cell surface area: (1) the cell membrane unfolding model and (2) the exocytosis model. In the former model, small microvilli or wrinkles on the cell surface act as membrane reservoirs and unfold to form the required membrane (Knutton et al., 1975; Erickson and Trinkaus, 1976; Figard and Sokac, 2014). In the latter model, exocytosis of intracellular vesicles supplies the necessary membrane (Schmoranzer et al., 2003; Bretscher, 2008). Migrating cells also need to change the cell surface area by extending and retracting the cell membrane; however, the cell membrane can physically stretch by 2–3% at the most (Mohandas and Evans, 1994). Expansion of the cell surface can be explained by either or both of the two models (Gauthier et al., 2011; Masters et al., 2013).

Endocytosis has been reported to be suppressed during cell division. Clathrin-mediated endocytosis (CME) is halted in dividing Hela cells (Fielding and Royle, 2013), although this is still controversial (Boucrot and Kirchhausen, 2007; Tacheva-Grigorova et al., 2013). Pinocytosis and phagocytosis are also suppressed during mitosis of macrophages and cultured animal cells (Berlin et al., 1978; Raucher and Sheetz, 1999). Suppression of endocytosis may be responsible for the increased cell surface area observed during cell division. Exocytosis also contributes to cytokinesis, although its quantitative information during cell division is unavailable (Goss and Toomre, 2008). Exocyst-dependent membrane addition is required for anaphase cell elongation and cytokinesis in *Drosophila* (Giansanti et al., 2015). In addition, exocytosis contributes to contraction of the cleavage furrow in yeast (Gerien and Wu, 2018) and *Xenopus* eggs (Straight and Field, 2000).

Recently, we showed that *Dictyostelium* cells could migrate by extending large pseudopods when flattened by pressing with an agar sheet; however, the cells exhibited neither wrinkles of the cell membrane nor thin extensions such as filopodia or microvilli. Therefore, even without membrane reservoir unfolding, the cells were able to migrate under the agar overlay (Tanaka et al., 2017). Under this condition, we could precisely measure the cell surface area, without influence of surface membrane reservoirs, and showed that the cell surface area was almost constant during cell migration. Furthermore, by staining the cell membrane with a fluorescent lipid analog, we have shown that the cell membrane is rapidly turned over by endocytosis and exocytosis, in a manner directly dependent on cell migration velocity (Tanaka et al., 2017).

Here, we measured the total cell surface area during cell division by the agar overlay method. Because the cells divide normally under an agar overlay, unfolding of the surface membrane reservoirs is not required for cell division. We found that the total cell surface area increased by about 20% through exocytosis during cytokinesis. The furrowing observed during cytokinesis was indispensable for the cell membrane increase, and vice versa. Both exocytosis and endocytosis are strictly regulated to control the cell shape change during cell division.

## Materials and Methods

### Cell Culture

*Dctyostelium discoideum* wild-type (AX2) cells and all mutant cells were cultured in HL5 medium (1.3% bacteriological peptone, 0.75% yeast extract, 85.5 mM D-glucose, 3.5 mM Na_2_HPO_4_, and 3.5 mM KH_2_PO_4_, pH 6.4) at 22°C. Cells were cultured in suspension at 150 rpm or on plastic dishes. To synchronize the cell cycle and increase the number of mitotic cells, cells were cultured at 10°C for 16 h and then treated with 100 μM TB at 22°C for 3.5 h. To start cell division, TB was removed by centrifugation and media exchange. HS1 cells were originally generated by Manstein et al. (1989). *Chc* null cells and temperature-sensitive *secA* mutant cells were originally generated by Ruscetti et al. (1994) and Zanchi et al. (2010), respectively.

### Plasmids and Transformation

Expression vectors containing GFP-ABD (actin-binding domain of filamin), GFP-alpha-tubulin, GFP-clathrin light chain (Fujimoto et al., 2019), or GFP-histone were transformed into AX2 cells by electroporation or laserporation as described previously (Yumura et al., 1995; Yumura, 2016). Positive cells were selected using 10 μg/mL G418 (Wako, Osaka, Japan).

### TB Treatment

To completely depolymerize the microtubules of cells at the interphase, the cells were incubated on ice for 30 min in the presence of 100 μM thiabendazole (TB, Tokyo Chemical Industry, Co. Ltd., Tokyo, Japan) and then transferred to incubation at 22°C. Empirically, depolymerization of microtubules in the interphase cells take longer (at least 2 h) at 22°C in the presence of TB. On the other hand, the microtubules in dividing cells quickly depolymerize, even with a lower concentration of TB (20 μM). To observe dividing cells after the application with TB, cells expressing GFP-tubulin were placed on a coverslip (18 mm × 18 mm, No. 1, Matsunami, Inc., Osaka, Japan) and overlaid with a thin agar sheet as described previously (Yumura et al., 1984). A small drop of TB solution was applied to the surface of the agar sheet at a final concentration of 20 μM prior to microscopy.

### Microscopy

Cells were placed on a coverslip and overlaid with an agar sheet. After the agar overlay, the cells expressing GFP-ABD were observed under an optical sectioning fluorescence microscope (Deltavision, GE Healthcare Life Science, United Kingdom). Z-axis images with an interval of 0.2–0.3 μm were acquired every 30 s. Each image was processed by deconvolution using the Deltavision system to remove out-of-focus images.

To normalize the cell division stage, MSI was used as described previously (Jahan and Yumura, 2017). MSI was computed from the long axis (L) and short axis (l), where the short axis represents the width of the furrow, using the following formula:

(1) MSI=(L-l)/L

When the MSI is 0, the cell is round; when the MSI is 1, cell division is completed.

Fixed cells were observed under a fluorescence microscope (TE 300, Nikon, Japan) equipped with regular ultraviolet (UV) and TRITC filter sets. Fluorescence images of live cells expressing GFP-tubulin or GFP-ABD, and membrane uptake in the presence of FM1-43 (Thermo Fisher Scientific, Tokyo, Japan), were observed using a confocal microscope (LSM510, Zeiss, Germany) at a time interval of 20–30 s.

Cells expressing GFP-clathrin (clathrin light chain) were observed using a custom-made TIRF microscope at a time interval of 2–5 s (Yumura et al., 2008). GFP-clathrin dots were counted manually by ImageJ software^1^. The lifetime of the GFP-clathrin dots was defined as the duration from their appearance to disappearance in the cell cortex.

### Measurement of the Cell Surface Area

To measure the cell surface area, we adopted two methods: (1) measurement from the cell outline and (2) measurement using a fluorescent lipid analog.

For the measurement from the cell outline, cells expressing GFP-histone or GFP-ABD were mildly pressed with an agar sheet, and phase-contrast and fluorescence images were captured with an interval of 0.5–1 min under an optical sectioning fluorescence microscope. The total cell surface areas were computed from the dorsoventral and lateral areas as described before (Tanaka et al., 2017). The lateral area of the cell was calculated from the outlines of cell in each optical z-section (7–10 slices). In some cases, we measured the cell surface area after fixation. For the fixation, agar-overlaid cells were fixed by immersing into ethanol containing 1% formaldehyde at −17°C and stained with DAPI (Sigma-Aldrich, Tokyo, Japan) and 50 ng/mL TRITC-conjugated phalloidin (Sigma-Aldrich), as previously described (Yumura et al., 1984).

For the fluorescence measurement using a fluorescent lipid analog, the partially-synchronized cells in suspension were stained with 10 μM FM1-43. Because the nutrient medium hampered the staining, the cells were stained after the medium had been exchanged with 15 mM Na/K phosphate buffer (pH 6.4) containing 0.1M sorbitol. The sorbitol was used to suppress the activities of contractile vacuoles (Zhu and Clarke, 1992). One minute after staining, the fluorescence intensity (excitation at 470 nm and emission at 570 nm) was measured by a fluorescence spectrophotometer (F-2500, Hitachi High-Technologies, Corp., Tokyo, Japan).

### Uptake of Cell Membranes

To measure the uptake of cell membranes in single cells, the cells were stained with the FM dye, and the fluorescence images were acquired over time by confocal microscopy. The fluorescence intensities of the cell membrane and the cell interior were calculated as previously described (Tanaka et al., 2017). Briefly, by using the ImageJ software, the integrated fluorescence intensity of a 1 μm-thick outline including the cell membrane was considered as the fluorescence of the cell membrane; the integrated fluorescence inside the outline was considered as the fluorescence of the cell interior.

### Scanning Electron Microscopy

The dividing cells were fixed and observed under a scanning electron microscope (SEM) (JSM-6360LA, JEOL, Ltd., Tokyo, Japan), as described previously (Tanaka et al., 2017).

### Statistical Analysis

Statistical analysis was performed using GraphPad Prism 7 (GraphPad Software, Inc., San Diego, CA, United States). Data are presented as the mean ± SD and analyzed using Student’s *t*-test for comparison between two groups, or by one-way ANOVA with Tukey’s multiple comparisons test.

## Results

### Unfolding of Surface Membrane Reservoirs Is Not Required for Cell Division

**FIGURE 1:**
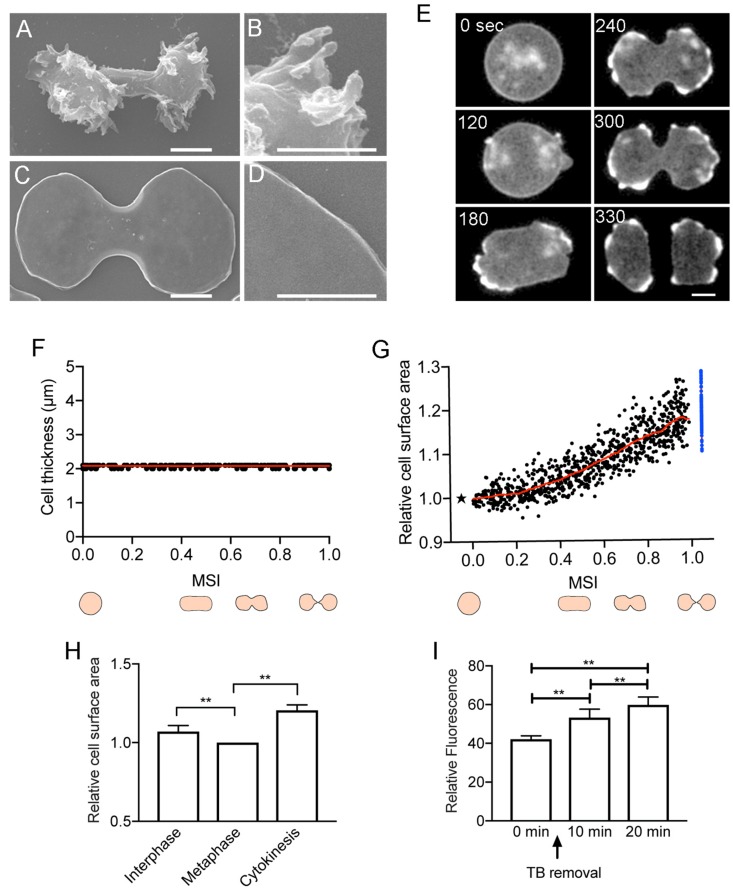
Total cell surface area during cell division. Typical scanning electron micrographs of dividing *Dictyostelium* cells without **(A,B)** and with the agar overlay **(C,D)**. Fixed cells were observed after the agar sheet was removed. **(B,D)** Show enlarged images of **(A,C)**. Bars: 5 μm. **(E)** Time course of cell division of a *Dictyostelium* cell expressing green fluorescent protein (GFP)- actin-binding domain of filamin (ABD). Bar: 10 μm. **(F)** Time course of the thickness of the agar-overlaid cells during cell division, measured by sectioning microscopy (*n* = 22). The cell division time was normalized according to the mitosis stage index (MSI). **(G)** Time course of the relative cell surface area during cytokinesis (versus MSI). The surface area is normalized to 1 at the metaphase (asterisk). The sum of the surface area of the two daughter cells is represented by blue dots. The red line shows the average. **(H)** Comparison of the relative surface area at the interphase, metaphase, and at end of cytokinesis. Data are presented as the mean ± SD and analyzed by one-way ANOVA with Tukey’s multiple comparisons test. **P ≤ 0.001, *n* = 15. **(I)** Relative fluorescence intensities of FM1-43-stained cells measured by a fluorescence spectrophotometry. The fluorescence intensity of the stained cells is shown before thiabendazole (TB) removal (0 min) as well as 10 and 20 min after the initiation of cell division. Data are presented as the mean ± SD and analyzed by one-way ANOVA with Tukey’s multiple comparisons test. **P ≤ 0.001, three different experiments.

Like animal cells, *Dictyostelium* cells vigorously change their shape during cell division. When entering the mitotic phase, the cells stop migrating, become spherical, elongate, and constrict the cleavage furrow to separate into two daughter cells. The total cell surface area should be altered during these morphological changes. However, the many projections and wrinkles on the cell surface complicate the accurate measurement of cell surface area. Here, to minimize the small projections and surface wrinkles, the cells were flattened, by pressing with an agar sheet, to expand the cell membrane. Without the agar overlay, the cells were 7–9 μm in thickness, and under the agar overlay, the cells flattened to a thickness of about 2 μm. Even under this condition, the cells divided normally. The surface of the fixed cells was observed using a SEM after removing the agar sheet. While the cells without the agar overlay had many wrinkles and projections on the surface (Figures 1A,B), the cells under the agar overlay had flattened shapes and no signs of wrinkles or projections (Figures 1C,D). Although there could be wrinkles or folds in the cell membrane that are beyond the resolution of SEM, we have not found any such minute wrinkles or folds even by transmission electron microscopy (Tanaka et al., 2017). Therefore, we concluded that the unfolding of the surface membrane reservoirs is dispensable for cell division.

### Total Cell Surface Area During Cell Division

To examine the total surface area of dividing cells, cells expressing green fluorescent protein (GFP)-actin-binding domain of filamin (ABD), a marker of actin filaments, or GFP-histone, were observed under an agar overlay by sectioning microscopy (only GFP-ABD images are shown; Figure 1E). The thickness of the cells under the agar overlay remained at about 2 μm during cell division (Figure 1F). Because the division time varied between cells, we used the mitosis stage index (MSI; calculated from the long axis and short axis) to normalize the cell division time (Jahan and Yumura, 2017). When the MSI is 0, the cell shape is round, corresponding to the metaphase; when the MSI is 1, the cell separates into two daughter cells. The total cell surface area was measured from the outline and thickness of the cells. Figure 1G shows the time course of relative total cell surface area changes from the cell rounding stage (MSI = 0, metaphase) to the completion of cell division (MSI = 1). The total cell surface area showed a subtle increase from the cell rounding stage (metaphase to anaphase) to the elongation stage (MSI = 0.4); thereafter, the surface area increased linearly by about 20% (19.1 ± 4.3%, *n* = 83), from the initiation of furrowing to the final cell separation (Figure 1G). We also compared the surface area between the interphase and metaphase cells, and the surface area decreased slightly during this transition (7.01 ± 3.89%, *n* = 15). Here, the total cell surface area of the interphase cells was measured immediately before the prophase (Figure 1H). In these experiments (Figures 1A–H), we used no inhibitor to synchronize the cell division as described below.

To further evaluate the increase in cell surface area, partially synchronized cells were stained with the FM1-43 dye, which is a cell-impermeable fluorescent lipid analog that emits fluorescence when inserted into the outer leaflet of the cell membrane. The fluorescence intensity of the stained cells was measured by a fluorescence spectrophotometer. To synchronize the cells, they were cultured at 10°C for 16 h and then arrested at the metaphase by treating with 100 μM thiabendazole (TB), a microtubule depolymerizer, at 22°C for 3.5 h (Fujimoto et al., 2019). After the removal of TB by centrifugation, most of the cells divided within 20 min. Figure 1I shows the relative fluorescence intensities of the stained cells before TB removal (0 min), as well as 10 and 20 min after the initiation of cell division. The fluorescence intensity increased by 30% (29.72 ± 7.01%, three different experiments) over that of the mitosis-arrested cells. This was more than what was observed using the agar overlay method; however, fluorescence spectrophotometry may lead to an overestimation of the surface area because membrane internalized by endocytosis is included in the measurement.

### Membrane Uptake Is Suppressed During Cell Division

**FIGURE 2:**
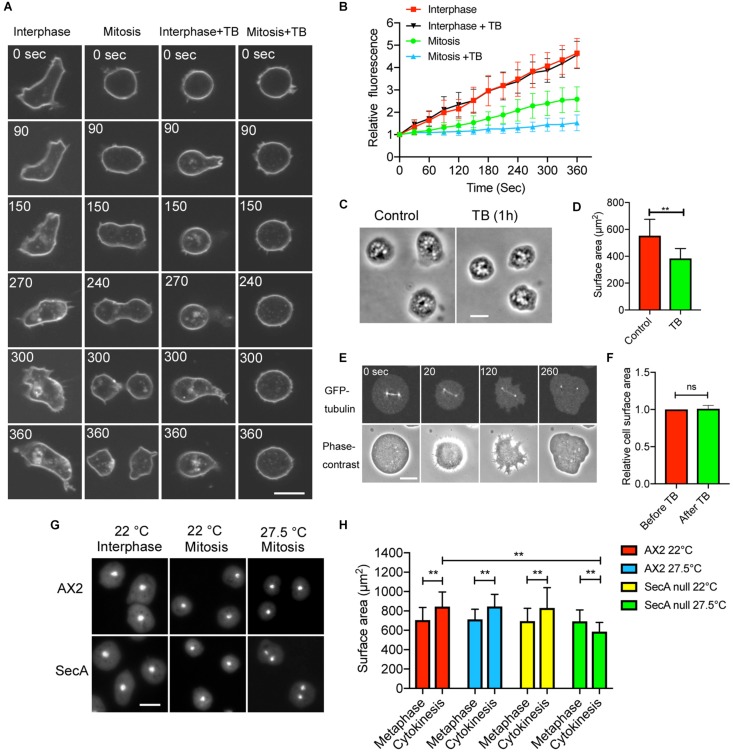
Membrane uptake is suppressed during cell division. **(A)** Time course of typical fluorescence images of cells at the interphase and during mitosis in the presence of FM1-43 (interphase, mitosis, interphase + TB, and mitosis + TB; TB: thiabendazole). In these experiments, the cells were stained without an agar overlay. Bar: 10 μm. **(B)** Time course of the fluorescence intensity of the inside (internalized membrane) of the cells stained with the FM dye in each condition (mean ± SD, *n* = 20). **(C)** Typical phase-contrast images of cells incubated before and 1 h after addition of TB. Bar: 10 μm. **(D)** Surface area of the cells in each condition. Data are presented as the mean ± SD and analyzed by Student’s *t*-test. **P ≤ 0.001, *n* ≥ 130. **(E)** Time course of fluorescence and phase-contrast images of the dividing cells expressing GFP-tubulin after the addition of TB. Because TB was added to the surface of the agar block, the cells were transiently raised (therefore, the cells shrunk slightly), but thereafter, the cells became flat. Note that the spindle disappeared 260 s after the addition of TB, and the cells were not able to undergo cytokinesis becoming binucleate. Bar: 10 μm. **(F)** Comparison of the surface area before and after addition of TB. Data are presented as the mean ± SD and analyzed by Student’s *t*-test. ns, not significant, *P* > 0.05, *n* = 44. **(G)** Typical fluorescence images of wild type (AX2) and *secA* null cells stained with 4′,6-diamidino-2-phenylindole (DAPI). Interphase cells cultured at 22°C, divided cells immediately after cytokinesis when cultured at 22°C, and divided cells immediately after cytokinesis when cultured at 27.5°C. *secA* null cells failed cytokinesis, resulting in binucleate cells at 27.5°C. Bar: 10 μm. **(H)** Comparison of the total cell surface area in wild type and *secA* null cells at the metaphase, cytokinesis (22°C), or failed cytokinesis (27.5°C). Here, we used TB for cells to be arrested at mitotic stage (Metaphase) and removed TB by media exchange to restart the cell division (Cytokinesis) at each temperature. Because the microtubule structures fully reappeared within 5 min, exocytosis was not affected with TB. Data are presented as the mean ± SD and analyzed by one-way ANOVA with Tukey’s multiple comparisons test. **P ≤ 0.001, *n* > 400.

Endocytosis is suppressed during cell division of cultured animal cells (Berlin et al., 1978; Raucher and Sheetz, 1999; Fielding and Royle, 2013; Aguet et al., 2016), which may explain the increase in cell surface area during cytokinesis. To examine the dynamics of the membrane uptake in dividing *Dictyostelium* cells, the cells were observed in the presence of the FM1-43 dye using confocal microscopy. Although the interphase cells vigorously internalized their membranes (Interphase, Figure 2A), mitotic cells showed only few internalized vesicles; during cytokinesis, the number of internalized vesicles gradually increased (Mitosis, Figure 2A). The fluorescence intensity time course for internalized membrane, shown in Figure 2B, indicates that membrane uptake is substantially suppressed by about 50%.

Microtubules play an important role in membrane trafficking. Interphase cells have approximately 30 microtubules elongating from a centriole associated with the nucleus. Mitotic cells have a mitotic spindle but no astral microtubules from the prophase to the early anaphase. At early anaphase, microtubules begin to elongate, reaching the cell cortex at the late anaphase (Kitanishi-Yumura and Fukui, 1987). In presence of TB, the membrane uptake was not suppressed in the interphase cells (Interphase + TB, Figures 2A,B). Interestingly, at interphase, cell size was reduced during the 1 h incubation with TB (Figure 2C), and the cell surface area was also significantly decreased (Figure 2D). It is plausible that the surface area reduction is caused by the TB-induced inhibition of (microtubule-dependent) exocytosis, while the ongoing endocytosis is unimpaired.

On the other hand, in cells under mitotic arrest, membrane uptake was substantially suppressed in the presence of TB (Mitosis + TB, Figures 2A,B), although some membrane uptake was observed. When TB was applied to the anaphase cells expressing GFP-tubulin, the mitotic spindle disappeared, leaving only centrosomes, and the cells failed cytokinesis without furrowing (Figure 2E). Interestingly, the total surface area did not change with TB treatment of the anaphase cells (Figure 2F), contrary to the result for the interphase cells. Therefore, it is plausible that TB impedes exocytosis because the endocytosis is stalled (Figure 2B), which suggests that the exocytosis is dependent on the microtubules in the mitotic phase as well as in the interphase.

To further clarify the contribution of exocytosis to the cell membrane increase, we used temperature-sensitive *secA* (encoding an exocytic protein) mutant cells. These cells show deficient exocytosis at 27.5°C (Zanchi et al., 2010). At the permissive temperature (22°C), cytokinesis proceeded normally, but at the restricted temperature, cytokinesis failed, and the cells became binucleate (Figure 2G). The total surface area did not increase in the cells without cytokinesis, contrary to what was observed at the permissive temperature (Figure 2H). Therefore, exocytosis is necessary for cytokinesis.

### Clathrin-Mediated Endocytosis Is Suppressed During Cell Division

**FIGURE 3:**
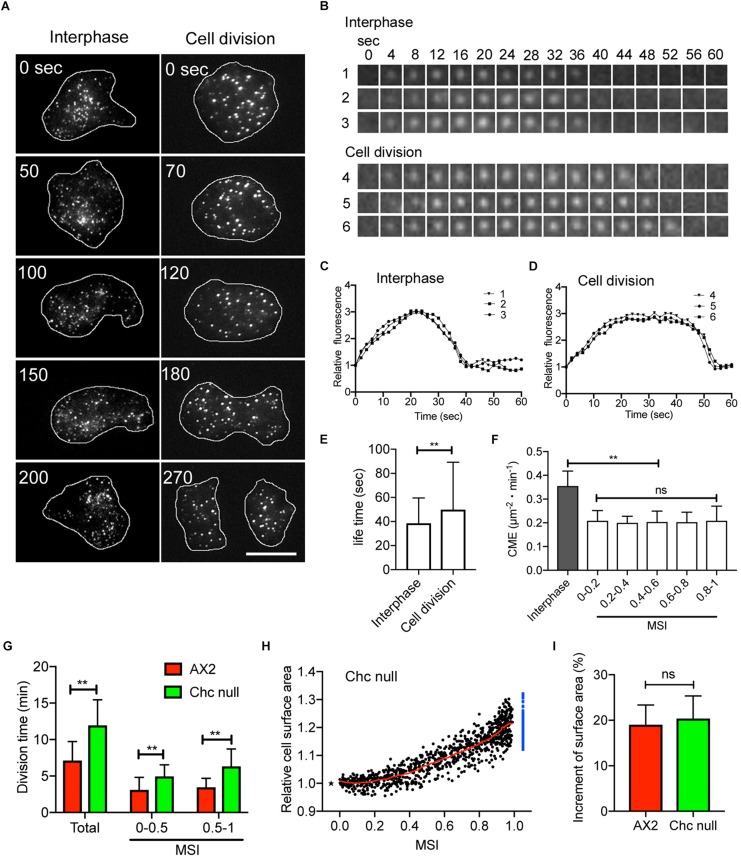
Clathrin-mediated endocytosis (CME) is suppressed during cell division. **(A)** Typical total internal reflection fluorescence (TIRF) microscopic images of cells expressing GFP-clathrin at the interphase and mitosis stage. **(B)** Time courses of typical TIRF images of clathrin dots in cells at the interphase (1, 2, and 3) and mitotic (4, 5, and 6) stages, respectively. **(C,D)** Time courses of fluorescence intensities of the clathrin dots in the cells at the interphase and mitotic stages **(B)**. **(E)** Comparison of the lifetime of clathrin dots between interphase and dividing cells. Data are presented as the mean ± SD and analyzed by Student’s *t*-test. **P < 0.001, *n* > 2,000. **(F)** CME in the interphase and during cell division (MSI 0–0.2, 0.2–0.4, 0.4–0.6, 0.6–0.8, and 0.8–1.0). Here, CME was counted as the number of disappearing dots per unit area and unit time (μm^–2^ min^–1^). **(G)** Comparison of division duration (Total, MSI 0–0.5, and MSI 0.5–1.0) between wild-type (AX2) and *clathrin heavy chain* (*chc*) null cells. Data are presented as the mean ± SD and analyzed by Student’s *t*-test. **P ≤ 0.001, *n* ≥ 53. **(H)** Changes in total cell surface area versus MSI during cytokinesis in *chc* null cells (*n* = 53). The surface area is normalized to 1 at the metaphase (asterisk). The sum of the surface area of the two daughter cells is represented by blue dots. The red line shows the average. **(I)** Comparison of the increase in cell surface area immediately before cytokinesis between wild type (AX2) and *chc* cells. Data are presented as the mean ± SD and analyzed by Student’s *t*-test. ns, not significant, *P* > 0.05, *n* ≥ 53.

The change in cell surface area during cell division in animal cells has been explained by suppression of CME (Fielding and Royle, 2013; Kaur et al., 2014; Aguet et al., 2016), although this is still controversial (Boucrot and Kirchhausen, 2007; Tacheva-Grigorova et al., 2013). We examined the contribution of CME on the surface area of dividing *Dictyostelium* cells by observing cells expressing GFP-clathrin under a total internal reflection fluorescence (TIRF) microscope. Many fluorescent dots, representing coated pits, were observed in the cell cortex of cells at the interphase and mitotic stages (Figure 3A), which confirmed previous observations (Macro et al., 2012; Fujimoto et al., 2019). Figure 3B shows a typical time course of individual dots that appeared and then disappeared in the cortex. Figures 3C,D show time courses of the fluorescence intensities of these dots in the interphase and mitotic cells, respectively. When dots disappear, endocytic vesicles are considered to be released from the cell membrane (Macro et al., 2012). Unlike the coated pits observed in the interphase cells, those in the dividing cells remained visible for a longer duration in the cortex. Figure 3E shows that the lifetime of the coated pits is significantly increased in the cells at the mitotic stage compared with the cells at the interphase (*n* > 2000 dots, each). Figure 3F shows that CME in the dividing cells is significantly suppressed at all MSIs compared with CME in the interphase cells. Here, CME was evaluated as the number of disappearing dots per unit area and unit time (μm^–2^ min^–1^).

Next, we examined the total cell surface area during cytokinesis in *clathrin heavy chain* (*chc*) null cells. Although *chc* null cells show defective cytokinesis in suspension culture (Niswonger and O’Halloran, 1997), most of the cells divide normally in the surface culture. We expected that the cell surface area increased faster in chc null cells than in wild type cells, thereby mutant cells divided faster. However, cell division was slower in the *chc* null cells. The duration increased overall relative to the furrowing (MSI of 0–0.5 and 0.5–1, respectively; Figure 3G). However, as shown in Figure 3H, the total surface area of the *chc* null cells increased by about 20% (20.39 ± 4.96%, *n* = 53), which is not significantly different from that observed in the wild type cells (Figure 3I). Therefore, although CME is suppressed during cell division, CME does not appear to contribute significantly to the regulation of cell surface area, which is contrary to previous reports in cultured animal cells (Kaur et al., 2014; Aguet et al., 2016).

### Proper Furrowing Is Required for the Surface Area Increase

**FIGURE 4:**
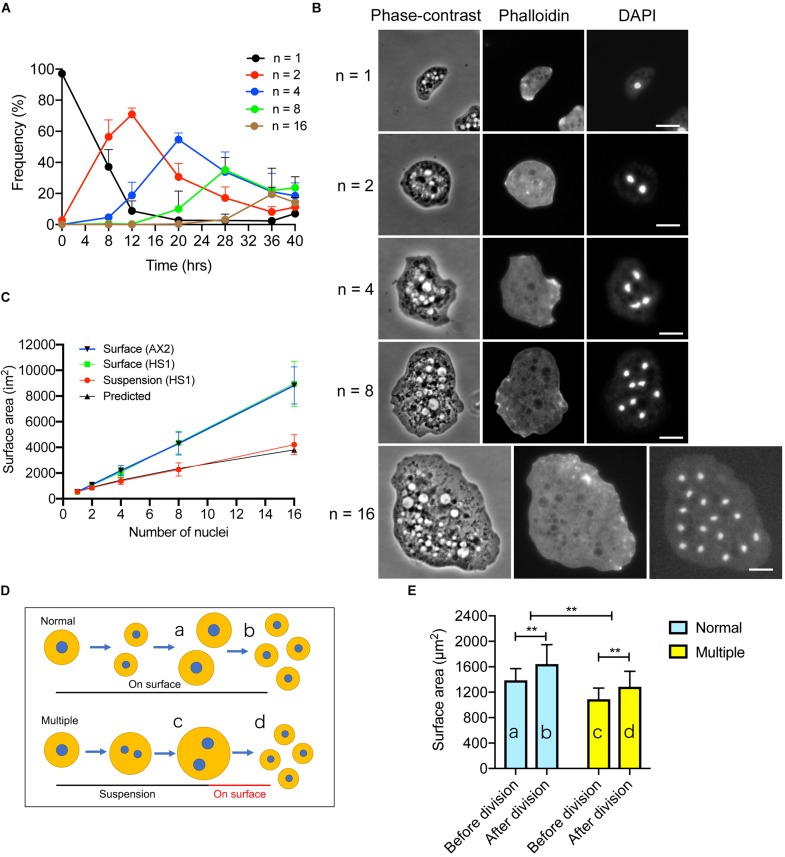
Proper furrowing is required for the increase in surface area. **(A)** Time course of multinucleation of myosin II null (HS1) cells in suspension culture. The frequencies of the cells having a single, 2, 4, 8, or 16 nuclei over time. Although the cells different numbers of nuclei (3, 5, etc.) gradually increased, only the cells with single, 2, 4, 8, and 16 nuclei are plotted (mean ± SD, *n* > 500). **(B)** Typical phase-contrast and fluorescence images of HS1 cells with single, 2, 4, 8, and 16 nuclei at each peak of the graph in **(A)**. The cells were overlaid with agar, fixed, and stained with tetramethylrhodamine isothiocyanate (TRITC) phalloidin and DAPI. Bars: 10 μm. **(C)** Surface area of HS1 cells plotted versus the number of nuclei when cells were cultured in suspension culture (Suspension HS1) and in surface culture (Surface HS1). The surface area of wild type (AX2) cells was also plotted versus the number of nuclei in surface culture (Surface AX2). Data are presented as the mean ± SD, *n* > 500. For the surface culture, the surface area was multiplied by the number of nuclei of the dividing cells such as 2, 4, 8, and 16. The predicted surface area (Predicted) is calculated by subtracting each area that should be incremented during the cytokinesis. **(D)** Scheme to explain the experiments. Multinucleate HS1 cells can divide in surface culture by binary fission (Normal). On the other hand, HS1 cells become multinucleate in suspension culture. The multinucleate cells can then divide by attaching to the surface (Multiple). The cell surface areas were compared before (a,c) and after (b,d) division. **(E)** The cell surface areas in the experiments shown in **(D)** are compared for Normal and Multiple. Data are presented as the mean ± SD and analyzed by Student’s *t*-test. **P ≤ 0.001, *n* > 130.

As described above, membrane surface area increase is necessary for cytokinesis; we therefore examined whether cytokinesis is required for the membrane surface area increase in *Dictyostelium*. Myosin II null (HS1) cells can divide by binary fission in surface culture depending on the opposite traction forces of the daughter halves (Neujahr et al., 1997; Jahan and Yumura, 2017). However, when cultured in suspension, the cell size increases and multiple nuclei are formed by normal nuclear division accompanying spindle formation (Zang et al., 1997; Taira and Yumura, 2017). As the culture duration increased, the number of nuclei increased by a power of two (e.g., 1, 2, 4, 8, and 16, Figure 4A). The doubling time of nuclei (about 8 hrs) was similar to the that of cell growth in wild type cells. Figure 4B shows typical phase-contrast and fluorescence microscopy images of multinucleate cells stained with tetramethylrhodamine isothiocyanate (TRITC)-conjugated phalloidin and 4′,6-diamidino-2-phenylindole (DAPI). The total surface area of the cells was measured after the agar overlay. When HS1 cells were cultured on an adhesive substratum, their cell surface areas increased linearly in a manner similar to wild type cells (Surface, Figure 4C: the surface area of a single cell was multiplied by the number of daughter cells, e.g., 2, 4, 8, or 16). However, the increase in surface area of cells in suspension culture (Suspension, Figure 4C) was not consistent with this observation; instead, the increase could be predicted by subtracting each area that should be incremented during each cytokinesis (Predicted, Figure 4C). Without furrowing (cytokinesis), about 20% of the membrane increment was lost. Therefore, we concluded that without proper furrowing (cytokinesis), the cell surface area could not increase.

Next, we examined the changes in the surface area upon cell division of multinucleate HS1 cells attached to the adhesive substratum. Within 1 h, the multinucleate cells divided into mononucleate cells, with multiple furrowing, by traction-mediated cytokinesis (Figures 4Dc,d). The cell surface area increased by about 20% after cytokinesis (Figure 4E, 18.04%, *n* > 130), in a manner similar to the increase observed during cytokinesis in wild type cells (Figures 4Da,b); however, the cell membrane increase did not fully recover to that of the constantly dividing cells on surface. Therefore, independent of the number of nuclei and size of the cells, approximately 20% of the membrane is added during this cytokinesis.

## Discussion

Here, we precisely measured the total cell surface area in dividing cells, flattened by the agar overlay method, during which the complex unfolding of surface membrane reservoirs is eliminated. Because the cells divide normally under this condition, unfolding of the surface membrane reservoirs is not required for cell division. Actually, without agar-overlay, the number of projections and wrinkles on the surface of dividing cells was similar to that of interphase cells as far as we observed using SEM (Tanaka et al., 2017).

**FIGURE 5:**
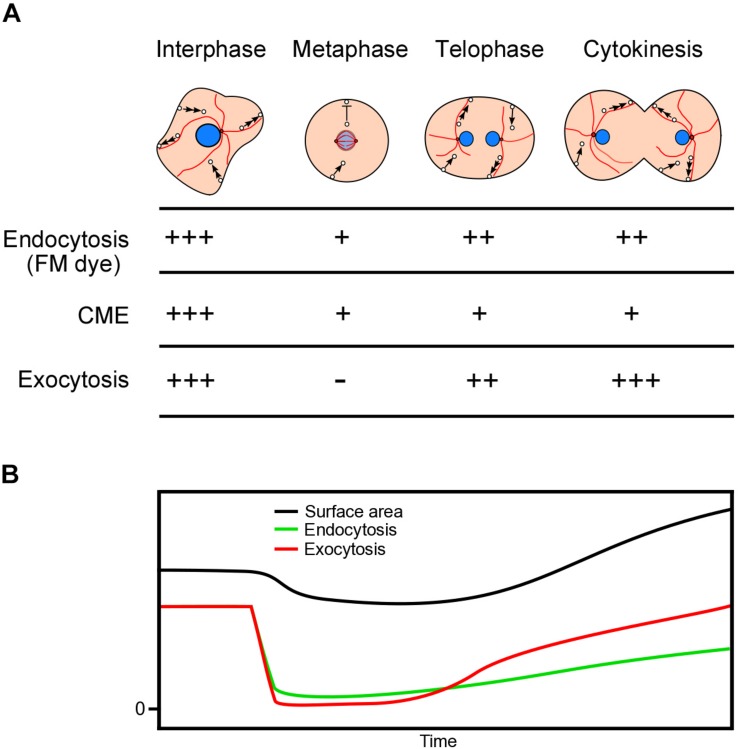
Dynamics of cell membrane surface area during cell division. **(A)** Summary of the dynamics of microtubules, endocytosis, and exocytosis during cell division. In the interphase cells, endocytosis, and exocytosis are balanced to maintain a constant total cell surface area. When the cells enter the prophase, astral microtubules disappear, resulting in the suppression of exocytosis. Exocytosis recovers as astral microtubules reappear during the telophase. Endocytosis is suppressed from the prophase to the metaphase and recovers by half during cytokinesis. CME is suppressed from the prophase to cytokinesis. **(B)** Time course of estimated endocytosis and exocytosis and the relative total cell surface area.

Using the agar overlay method, we found that the total cell surface area slightly decreased from the interphase to the metaphase, and then increased by about 20% during cytokinesis. The cell surface area seems to be strictly regulated both by endocytosis and exocytosis. Figure 5 shows a summary of the estimated endocytosis and exocytosis dynamics during cell division. In the interphase cells, endocytosis and exocytosis are balanced to maintain a constant total cell surface area. When entering the mitotic phase, astral microtubules disappear, resulting in suppression of exocytosis due to suspension of microtubule-dependent membrane trafficking. In addition, endocytosis is partially suppressed. Therefore, the total surface area begins to decrease, which may contribute to cell rounding. After the telophase, as astral microtubules reach the cell cortex, the exocytosis and endocytosis recover. During cytokinesis, to increase the total cell surface area, exocytosis should exceed endocytosis.

Based on our previous studies using the agar overlay method, the total cell surface area does not change during cell migration, but the total cell membrane is refreshed with a half-life of 5 min via turnover by endocytosis and exocytosis (Tanaka et al., 2017). Because the membrane uptake of the cytokinetic cells was half of that observed in the interphase cells (Figure 2B), the cells should take up 25% of the cell membrane during cytokinesis (5 min). If exocytosis fully recovers as the astral microtubules elongate, it can add 50% of the cell membrane. However, visualization of exocytosis during cell division is required in the future; currently there are no available tools to visualize exocytosis in *Dictyostelium* cells.

Based on the HS1 cell experiments, cytokinetic furrowing is required for the increase in the cell surface area, which is independent of proper spindle formation and nuclear division. Conversely, from the experiments using the *secA* null mutant, the increase in the cell surface is critical for cytokinesis. Previous studies have reported that the delivery of intracellular membrane vesicles to the cell membrane is required for constriction of the cleavage furrow in zebrafish, *C. elegans* embryos, *Drosophila* spermatocytes, and yeast (Li et al., 2006; Robinett et al., 2009; Giansanti et al., 2015; Wang et al., 2016; Kumar et al., 2019). As a candidate of the membrane vesicles source, Golgi-derived vesicles, lysosome, or endosome has been reported (Arden et al., 2007; Boucrot and Kirchhausen, 2007; Goss and Toomre, 2008; Montagnac et al., 2009). Although exocytosis is required for the increase of surface area in cytokinesis, it should be emphasized that the regulation of endocytosis also contributes to the regulation of the cell surface area.

The mechanism underlying suppression of membrane uptake during mitosis has been studied previously; however, these studies focused mainly on the contribution of membrane uptake for cell rounding. Three models for suppression of membrane uptake have been proposed. (1) Phosphorylation of epsin, a clathrin-adaptor protein, blocks the invagination of coated pits during mitosis (Chen et al., 1999). Although phosphorylation of epsin orthologs in *Dictyostelium* has not been reported, cells deficient in epsin have reduced CME (Brady et al., 2010). (2) An increase in membrane tension inhibits endocytosis during mitosis in animal cells (Raucher and Sheetz, 1999). The increase in membrane tension has also been reported to increase during mitosis in *Dictyostelium* cells (Srivastava et al., 2016). (3) Clathrin localizes primarily at the mitotic spindle to stabilize the structure and does not participate in endocytosis in dividing cultured animal cells (Royle et al., 2005). In *Dictyostelium* cells, we have not observed clathrin at the spindle. In *Dictyostelium* cells, clathrin evenly localizes in the cell cortex, whereas dynamin is known to localize at the cleavage furrow (Masud Rana et al., 2013; Fujimoto et al., 2019).

We favor the membrane tension model. As the astral microtubules disappear, exocytosis is suppressed and endocytosis proceeds at a low level, resulting in a reduced cell surface area. This reduction in the cell surface area increases the cell surface tension, resulting in cell rounding, and an increase in the tension beyond a critical level inhibits endocytosis. During cytokinesis, as the astral microtubules elongate, exocytosis recovers, and the membrane tension decreases, resulting in recovered endocytosis.

The contribution of CME to membrane dynamics during cell division is still controversial. CME does not change in dividing Hela or BSC1 cells (Boucrot and Kirchhausen, 2007). On the other hand, CME is inhibited at the early mitotic phase in human breast cancer cells (Aguet et al., 2016). In the present study, we found that CME remained suppressed during the entire cell division, whereas the total membrane uptake recovered during cytokinesis (FM dye experiments). The regulation of CME at the molecular level during cell division should be clarified in future experiments.

If the CME vesicles are 0.1 μm in diameter, and 1,400 vesicles are internalized during cell division, about 4% of the total cell membrane would be internalized. The suppression of CME is not sufficient for the observed membrane increase. Therefore, CME is not a key contributor to the suppression of the total membrane uptake. Incidentally, we could not find any gene homologous to caveolin in the *Dictyostelium* genome that would account for caveola-mediated endocytosis (Fujimoto et al., 2019). Presumably, other endocytosis mechanisms, such as macro-pinocytosis, may also be suppressed.

## Conclusion

Unfolding of surface membrane reservoirs was not required for cell division. The total cell surface area increased by 20% during cytokinesis. This increase was due to the imbalance between exocytosis and endocytosis. CME was significantly suppressed during cell division but did not significantly contribute to the cell surface area dynamics, contrary to previous reports. The furrowing observed during cytokinesis was indispensable for the increase in the cell membrane, and vice versa. Both exocytosis and endocytosis are strictly regulated to control the cell shape changes during cell division.

## Data Availability Statement

The datasets generated for this study are available on request to the corresponding author.

## Author Contributions

MT and KF were involved in the experimental work and data analysis. SY was involved in the project planning and data analysis. MT, KF, and SY wrote the manuscript.

## Conflict of Interest

The authors declare that the research was conducted in the absence of any commercial or financial relationships that could be construed as a potential conflict of interest.

## Footnotes

1 http://rsb.info.nih.gov/ij

## References

[B1] Aguet F. Upadhyayula S. Gaudin R. Chou Y. Y. Cocucci E. He K. (2016). Membrane dynamics of dividing cells imaged by lattice light-sheet microscopy. *Mol. Biol. Cell* 27 3418–3435. 27535432 10.1091/mbc.E16-03-0164 PMC5221578

[B2] Arden S. D. Puri C. Au J. S. Kendrick-Jones J. Buss F. (2007). Myosin VI is required for targeted membrane transport during cytokinesis. *Mol. Biol. Cell* 18 4750–4761. 17881731 10.1091/mbc.E07-02-0127 PMC2096599

[B3] Berlin R. D. Oliver J. M. Walter R. J. (1978). Surface functions during mitosis I: phagocytosis, pinocytosis and mobility of surface-bound Con A. *Cell* 15 327–341. 719746 10.1016/0092-8674(78)90002-8

[B4] Boucrot E. Kirchhausen T. (2007). Endosomal recycling controls plasma membrane area during mitosis. *Proc. Natl. Acad. Sci. U.S.A.* 104 7939–7944. 17483462 10.1073/pnas.0702511104 PMC1876551

[B5] Brady R. J. Damer C. K. Heuser J. E. O’Halloran T. J. (2010). Regulation of Hip1r by epsin controls the temporal and spatial coupling of actin filaments to clathrin-coated pits. *J. Cell Sci.* 123 3652–3661. 20923836 10.1242/jcs.066852 PMC2964106

[B6] Bretscher M. S. (2008). Exocytosis provides the membrane for protrusion, at least in migrating fibroblasts. *Nat. Rev. Mol. Cell Biol.* 9:916. 10.1038/nrm2419-c3 18946479

[B7] Cadart C. Monnier S. Grilli J. Saez P. J. Srivastava N. Attia R. (2018). Size control in mammalian cells involves modulation of both growth rate and cell cycle duration. *Nat. Commun.* 9:3275. 10.1038/s41467-018-05393-0 PMC6095894 30115907

[B8] Chen H. Slepnev V. I. Di Fiore P. P. De Camilli P. (1999). The interaction of epsin and Eps15 with the clathrin adaptor AP-2 is inhibited by mitotic phosphorylation and enhanced by stimulation-dependent dephosphorylation in nerve terminals. *J. Biol. Chem.* 274 3257–3260. 9920862 10.1074/jbc.274.6.3257

[B9] Erickson C. A. Trinkaus J. P. (1976). Microvilli and blebs as sources of reserve surface membrane during cell spreading. *Exp. Cell Res.* 99 375–384. 1269533 10.1016/0014-4827(76)90595-4

[B10] Fielding A. B. Royle S. J. (2013). Mitotic inhibition of clathrin-mediated endocytosis. *Cell Mol. Life Sci.* 70 3423–3433. 23307073 10.1007/s00018-012-1250-8 PMC3939358

[B11] Figard L. Sokac A. M. (2014). A membrane reservoir at the cell surface: unfolding the plasma membrane to fuel cell shape change. *Bioarchitecture* 4 39–46. 24844289 10.4161/bioa.29069 PMC4199810

[B12] Fujimoto K. Tanaka M. Rana A. Y. K. M. M. Jahan M. G. S. Itoh G. Tsujioka M. (2019). Dynamin-like protein B of *Dictyostelium* contributes to cytokinesis cooperatively with other dynamins. *Cells* 8:781. 10.3390/cells8080781 PMC6721605 31357517

[B13] Gauthier N. C. Fardin M. A. Roca-Cusachs P. Sheetz M. P. (2011). Temporary increase in plasma membrane tension coordinates the activation of exocytosis and contraction during cell spreading. *Proc. Natl. Acad. Sci. U.S.A.* 108 14467–14472. 21808040 10.1073/pnas.1105845108 PMC3167546

[B14] Gerien K. S. Wu J. Q. (2018). Molecular mechanisms of contractile-ring constriction and membrane trafficking in cytokinesis. *Biophys. Rev.* 10 1649–1666. 30448943 10.1007/s12551-018-0479-3 PMC6297088

[B15] Giansanti M. G. Vanderleest T. E. Jewett C. E. Sechi S. Frappaolo A. Fabian L. (2015). Exocyst-dependent membrane addition is required for anaphase cell elongation and cytokinesis in *Drosophila*. *PLoS Genet.* 11:e1005632. 10.1371/journal.pgen.1005632 26528720 PMC4631508

[B16] Goss J. W. Toomre D. K. (2008). Both daughter cells traffic and exocytose membrane at the cleavage furrow during mammalian cytokinesis. *J. Cell Biol.* 181:1047. 10.1083/jcb.200712137 PMC2442215 18573914

[B17] Guillou L. Babataheri A. Saitakis M. Bohineust A. Dogniaux S. Hivroz C. (2016). T-lymphocyte passive deformation is controlled by unfolding of membrane surface reservoirs. *Mol. Biol. Cell* 27 3574–3582. 27605708 10.1091/mbc.E16-06-0414 PMC5221589

[B18] Jahan M. G. S. Yumura S. (2017). Traction force and its regulation during cytokinesis in *Dictyostelium* cells. *Eur. J. Cell Biol.* 96 515–528. 28633918 10.1016/j.ejcb.2017.06.004

[B19] Kaur S. Fielding A. B. Gassner G. Carter N. J. Royle S. J. (2014). An unmet actin requirement explains the mitotic inhibition of clathrin-mediated endocytosis. *Elife* 3:e00829. 10.7554/eLife.00829 PMC3924242 24550251

[B20] Kitanishi-Yumura T. Fukui Y. (1987). Reorganization of microtubules during mitosis in *Dictyostelium*: dissociation from MTOC and selective assembly/disassembly in situ. *Cell Motil. Cytoskeleton* 8 106–117.

[B21] Knutton S. Sumner M. C. Pasternak C. A. (1975). Role of microvilli in surface changes of synchronized P815Y mastocytoma cells. *J. Cell Biol.* 66 568–576. 1158972 10.1083/jcb.66.3.568 PMC2109462

[B22] Kumar H. Pushpa K. Kumari A. Verma K. Pergu R. Mylavarapu S. V. S. (2019). The exocyst complex and Rab5 are required for abscission by localizing ESCRT III subunits to the cytokinetic bridge. *J. Cell Sci.* 132:jcs226001. 10.1242/jcs.226001 PMC6679584 31221728

[B23] Li W. M. Webb S. E. Lee K. W. Miller A. L. (2006). Recruitment and SNARE-mediated fusion of vesicles in furrow membrane remodeling during cytokinesis in zebrafish embryos. *Exp. Cell Res.* 312 3260–3275. 16876784 10.1016/j.yexcr.2006.06.028

[B24] Macro L. Jaiswal J. K. Simon S. M. (2012). Dynamics of clathrin-mediated endocytosis and its requirement for organelle biogenesis in *Dictyostelium*. *J. Cell Sci.* 125:5721. 10.1242/jcs.108837 PMC3575707 22992464

[B25] Manstein D. J. Titus M. A. De Lozanne A. Spudich J. A. (1989). Gene replacement in *Dictyostelium*: generation of myosin null mutants. *EMBO J.* 8 923–932. 2721503 10.1002/j.1460-2075.1989.tb03453.x PMC400892

[B26] Masters T. A. Pontes B. Viasnoff V. Li Y. Gauthier N. C. (2013). Plasma membrane tension orchestrates membrane trafficking, cytoskeletal remodeling, and biochemical signaling during phagocytosis. *Proc. Natl. Acad. Sci. U.S.A.* 110 11875–11880. 23821745 10.1073/pnas.1301766110 PMC3718161

[B27] Masud Rana A. Y. Tsujioka M. Miyagishima S. Ueda M. Yumura S. (2013). Dynamin contributes to cytokinesis by stabilizing actin filaments in the contractile ring. *Genes Cells.* 18 621–635. 23679940 10.1111/gtc.12060

[B28] Mohandas N. Evans E. (1994). Mechanical properties of the red cell membrane in relation to molecular structure and genetic defects. *Annu. Rev. Biophys. Biomol. Struct.* 23 787–818. 7919799 10.1146/annurev.bb.23.060194.004035

[B29] Montagnac G. Sibarita J. B. Loubery S. Daviet L. Romao M. Raposo G. (2009). ARF6 interacts with JIP4 to control a motor switch mechanism regulating endosome traffic in cytokinesis. *Curr. Biol.* 19 184–195. 19211056 10.1016/j.cub.2008.12.043

[B30] Neujahr R. Heizer C. Gerisch G. (1997). Myosin II-independent processes in mitotic cells of *Dictyostelium discoideum*: redistribution of the nuclei, re-arrangement of the actin system and formation of the cleavage furrow. *J. Cell Sci.* 110 123–137. 9044043 10.1242/jcs.110.2.123

[B31] Niswonger M. L. O’Halloran T. J. (1997). A novel role for clathrin in cytokinesis. *Proc. Natl. Acad. Sci. U.S.A.* 94 8575–8578. 9238018 10.1073/pnas.94.16.8575 PMC23021

[B32] Raucher D. Sheetz M. P. (1999). Membrane expansion increases endocytosis rate during mitosis. *J. Cell Biol.* 144 497–506. 9971744 10.1083/jcb.144.3.497 PMC2132908

[B33] Robinett C. C. Giansanti M. G. Gatti M. Fuller M. T. (2009). TRAPPII is required for cleavage furrow ingression and localization of Rab11 in dividing male meiotic cells of *Drosophila*. *J. Cell Sci.* 122 4526–4534. 19934220 10.1242/jcs.054536 PMC2787463

[B34] Royle S. J. Bright N. A. Lagnado L. (2005). Clathrin is required for the function of the mitotic spindle. *Nature* 434 1152–1157. 15858577 10.1038/nature03502 PMC3492753

[B35] Ruscetti T. Cardelli J. A. Niswonger M. L. O’Halloran T. J. (1994). Clathrin heavy chain functions in sorting and secretion of lysosomal enzymes in *Dictyostelium discoideum*. *J. Cell Biol.* 126 343–352. 8034739 10.1083/jcb.126.2.343 PMC2200034

[B36] Schmid-Schonbein G. W. Shih Y. Y. Chien S. (1980). Morphometry of human leukocytes. *Blood* 56 866–875. 6775712

[B37] Schmoranzer J. Kreitzer G. Simon S. M. (2003). Migrating fibroblasts perform polarized, microtubule-dependent exocytosis towards the leading edge. *J. Cell Sci.* 116 4513–4519. 14576345 10.1242/jcs.00748

[B38] Srivastava V. Iglesias P. A. Robinson D. N. (2016). Cytokinesis: robust cell shape regulation. *Semin. Cell Dev. Biol.* 53 39–44. 26481973 10.1016/j.semcdb.2015.10.023 PMC4837095

[B39] Straight A. F. Field C. M. (2000). Microtubules, membranes and cytokinesis. *Curr. Biol.* 10 R760–R770. 11069103 10.1016/s0960-9822(00)00746-6

[B40] Tacheva-Grigorova S. K. Santos A. J. Boucrot E. Kirchhausen T. (2013). Clathrin-mediated endocytosis persists during unperturbed mitosis. *Cell Rep.* 4 659–668. 23954786 10.1016/j.celrep.2013.07.017 PMC3849811

[B41] Taira R. Yumura S. (2017). A novel mode of cytokinesis without cell-substratum adhesion. *Sci. Rep.* 7:17694. 10.1038/s41598-017-17477-w PMC5735089 29255156

[B42] Tanaka M. Kikuchi T. Uno H. Okita K. Kitanishi-Yumura T. Yumura S. (2017). Turnover and flow of the cell membrane for cell migration. *Sci. Rep.* 7:12970. 10.1038/s41598-017-13438-5 PMC5636814 29021607

[B43] Varsano G. Wang Y. Wu M. (2017). Probing mammalian cell size homeostasis by channel-assisted cell reshaping. *Cell Rep.* 20 397–410. 28700941 10.1016/j.celrep.2017.06.057

[B44] Wang N. Lee I. J. Rask G. Wu J. Q. (2016). Roles of the TRAPP-II complex and the exocyst in membrane deposition during fission yeast cytokinesis. *PLoS Biol.* 14:e1002437. 10.1371/journal.pbio.1002437 27082518 PMC4833314

[B45] Yumura S. (2016). A novel low-power laser-mediated transfer of foreign molecules into cells. *Sci. Rep.* 6:22055. 10.1038/srep22055 PMC4763237 26902313

[B46] Yumura S. Matsuzaki R. Kitanishi-Yumura T. (1995). Introduction of macromolecules into living *Dictyostelium* cells by electroporation. *Cell Struct. Funct.* 20 185–190. 7586008 10.1247/csf.20.185

[B47] Yumura S. Mori H. Fukui Y. (1984). Localization of actin and myosin for the study of ameboid movement in *Dictyostelium* using improved immunofluorescence. *J. Cell Biol.* 99 894–899. 6381508 10.1083/jcb.99.3.894 PMC2113401

[B48] Yumura S. Ueda M. Sako Y. Kitanishi-Yumura T. Yanagida T. (2008). Multiple mechanisms for accumulation of myosin II filaments at the equator during cytokinesis. *Traffic* 9 2089–2099. 18939956 10.1111/j.1600-0854.2008.00837.x

[B49] Zanchi R. Howard G. Bretscher M. S. Kay R. R. (2010). The exocytic gene secA is required for *Dictyostelium* cell motility and osmoregulation. *J. Cell Sci.* 123 3226–3234. 20807800 10.1242/jcs.072876 PMC2939799

[B50] Zang J. H. Cavet G. Sabry J. H. Wagner P. Moores S. L. Spudich J. A. (1997). On the role of myosin-II in cytokinesis: division of *Dictyostelium* cells under adhesive and nonadhesive conditions. *Mol. Biol. Cell* 8 2617–2629. 9398680 10.1091/mbc.8.12.2617 PMC25732

[B51] Zhu Q. Clarke M. (1992). Association of calmodulin and an unconventional myosin with the contractile vacuole complex of *Dictyostelium discoideum*. *J. Cell Biol.* 118 347–358. 1629238 10.1083/jcb.118.2.347 PMC2290049

